# Anthocyanins: Factors Affecting Their Stability and Degradation

**DOI:** 10.3390/antiox10121967

**Published:** 2021-12-09

**Authors:** Bianca Enaru, Georgiana Drețcanu, Teodora Daria Pop, Andreea Stǎnilǎ, Zorița Diaconeasa

**Affiliations:** Faculty of Food Science and Technology, University of Agricultural Science and Veterinary Medicine, 400372 Cluj-Napoca, Romania; bianca.enaru@stud.ubbcluj.ro (B.E.); georgiana.dretcanu@stud.ubbcluj.ro (G.D.); teodora-daria.pop@student.usamvcluj.ro (T.D.P.); andreea.stanila@usamvcluj.ro (A.S.)

**Keywords:** anthocyanins, stability, degradation, pigments, health benefit

## Abstract

Anthocyanins are secondary metabolites and water-soluble pigments belonging to the phenolic group, with important functions in nature such as seed dispersal, pollination and development of plant organs. In addition to these important roles in plant life, anthocyanins are also used as natural pigments in various industries, due to the color palette they can produce from red to blue and purple. In addition, recent research has reported that anthocyanins have important antioxidant, anticancer, anti-inflammatory and antimicrobial properties, which can be used in the chemoprevention of various diseases such as diabetes, obesity and even cancer. However, anthocyanins have a major disadvantage, namely their low stability. Thus, their stability is influenced by a number of factors such as pH, light, temperature, co-pigmentation, sulfites, ascorbic acid, oxygen and enzymes. As such, this review aims at summarizing the effects of these factors on the stability of anthocyanins and their degradation. From this point of view, it is very important to be precisely aware of the impact that each parameter has on the stability of anthocyanins, in order to minimize their negative action and subsequently potentiate their beneficial health effects.

## 1. Introduction

Anthocyanins are a class of natural water-soluble pigments that are part of the flavonoid family. They are very widespread in nature, found not only in the colored petals of flowers but also in the roots, stems, tubers, leaves, fruits and seeds [1,2]. This type of pigment has a strong absorption in the UV-visible region of the electromagnetic spectrum and is the main determinant of red-blue colors and their derivatives in the plant kingdom. These characteristics place them second in importance, immediately following chlorophyll pigments [3]. Anthocyanins play an important role in seed dispersal, pollination, development of plant organs, but also in their adaptation to various changes in biotic (pathogenic attacks) and abiotic (drought, lack of nutrients, high intensity light) [4] factors. Due to their chemical structure, with a central core in the form of 2-phenylbenzopyrylium or flavylium cation, anthocyanins can be classified as polyphenols and secondary metabolites [5].

**Figure 1:**
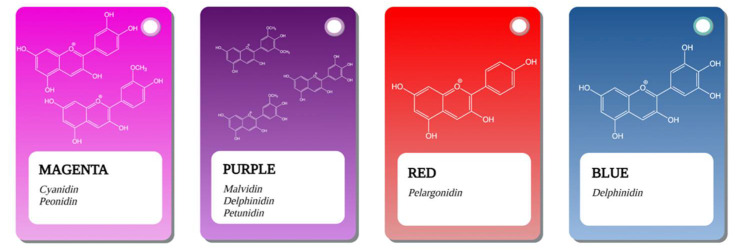
The structure and color of the most common anthocyanins present in nature.

Structurally, anthocyanins are found in the form of glycosides of polyhydroxy and polymethoxy derivatives of 2-phenylbenzopyrrile salts and are composed of an aglycone also called anthocyanidin and a carbohydrate residue that may be glucose, xylose, galactose, arabinose, rhamnose or rutinose. These carbohydrate residues are generally attached to the anthocyanidin skeleton through the C3 hydroxyl group in ring C [6,7]. Organic acids can be added to the sugar moieties and thus the acylation of the initial structure takes place. The most common acylation compounds include aromatic acids such as p-coumaric, sinapic, gallic, ferulic and caffeic acid, but also a number of aliphatic acids such as the malic, succinic, oxalic, tartaric and acetic acid [8,9]. It should be noted that this structure makes them dependent on the composition and conditions of the solution in which they are dissolved. Additionally, anthocyanins can perform interactions with other compounds but also with each other, where both their color and their structural balance are influenced [10]. In this way, a class of compounds comprising over 700 distinct species of anthocyanins is obtained, but only 6 of them (cyanidin, pelargonidin, delphinidin, peonidin, petunidin and malvidin) are abundantly found in nature and represent about 90% of all anthocyanins identified so far [11]. These 6 anthocyanins are found in fruits and vegetables in various percentages, for example: 50%—cyanidin, 12%—in the case of pelargonidin, delphinine, petunidin and 7%—for peonidin and malvidin. In nature, cyanidin is found predominantly in berries and other red vegetables because it appears in the form of a red-purple pigment, similar to the color magenta. Pelargonidin appears freely in the form of a red pigment but gives flowers an orange hue and fruits a red color. Delphinidin appears as a blue-reddish or purple pigment in the plant, leading to the blue color of flowers. Petunidin is a water-soluble methylated anthocyanin, dark red or purple, which is often found in blackcurrants and purple flowers. Peonidin is another methylated anthocyanin, which appears in the form of a magenta pigment, also found abundantly in berries, grapes and in red wines. Malvidin is an O-methylated anthocyanin, which appears as a purple pigment, and determines the blue color of certain flowers, but it is the major pigment in red wines [12]. Thus, the anthocyanins cyanidin, pelargonidin and delphinidin are often found in fruits, while in flowers the predominant anthocyanins are peonidin, petunidin and malvidin [13]. All these color variations of anthocyanins are represented in Figure 1.

**Figure 2:**
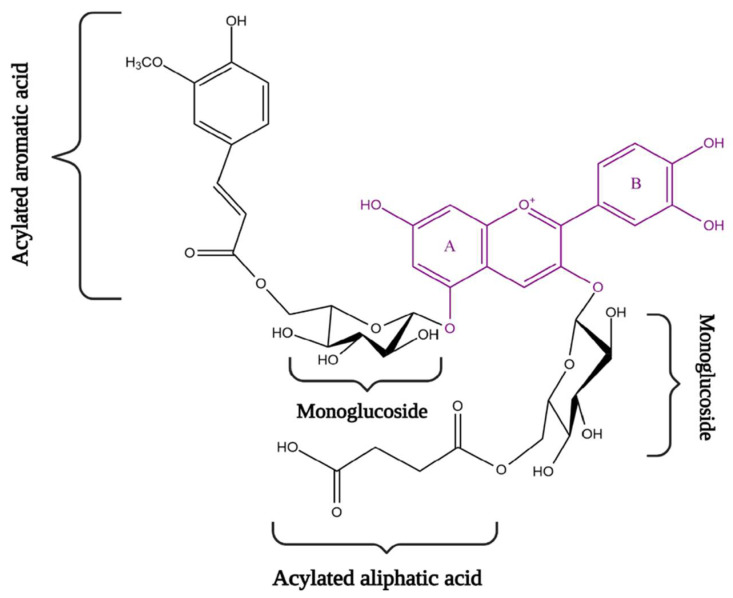
General chemical structure of anthocyanidin (flavylium cation) with sugar moieties and aromatic or aliphatic acid.

Therefore, Figure 2 shows the structural variations in anthocyanins, which occur due to differences in the type of sugar, position and number of hydroxyl or methoxyl groups in the B ring but also due to the presence or absence of acylation with both aromatic and aliphatic compounds [8]. The chemical structure of these polyphenols appears in the form of a C6 skeleton, and is pH dependent [14,15,16].

Anthocyanin synthesis takes place in a special segment of the flavonoid synthesis pathway, which is regulated on several levels. The first step is the conversion of phenylalanine to cinnamic acid, and subsequently, it is transformed into the main precursor of anthocyanins, 4-coumaroyl CoA, through a series of reactions catalyzed by cinnamate 4-hydroxylase (C4H) and 4-coumaroyl CoA ligase (4CL). In the next step one molecule of 4-coumaroyl CoA and three molecules of malonyl CoA will be condensed using the chalcone synthase, resulting in chalcones. Finally, after a series of enzymatic reactions, the main anthocyanins will be produced [17]. This synthesis takes place in the cytosol, and the anthocyanins obtained will be transported to vacuoles, where they will be stored as colored coalescences called anthocyanic vacuolar inclusions [18].

In addition, recent research has concluded that the synthesis and accumulation of anthocyanins can be controlled by regulating the expression of certain genes, epigenetic changes in plants, and post-translational changes in proteins that coordinate the activity of the transcription factor [17]. Furthermore, the regulatory molecules (precursors and enzymes) of biosynthetic and degradation pathways influence the amount of anthocyanin in different plant regions and in different plants [19].

Both red and blue fruits and vegetables are the main sources of anthocyanins, but their anthocyanin content suffers from important variations between different species, and within the same species. The factors influencing anthocyanin content include plant variety, climate, growing area, cultivation processes, harvesting period, ripening, seasonal variability, processing and storage, light and temperature [20].

Initially these compounds were mainly used as natural dyes in the food industry, due to their wide range of colors, ranging from salmon pink to red and from purple to almost black [21]. Over the past few years, numerous studies have reported that anthocyanins exhibit anti-cancer, anti-inflammatory, antimicrobial and antioxidant properties, with a role in reducing the incidence of cardiovascular disease, diabetes and obesity [22]. Therefore, it can be stated that anthocyanins have enormous potential; they can be used both in food science and in the medical field [23].

The main disadvantage of anthocyanins is their extremely low stability, which is easily influenced by a wide range of parameters such as relative humidity, light, pH, temperature, sugars (acylated and unacylated), vitamin C, oxygen levels, sulfur dioxide or sulfites, enzymes, co-pigments and metal ions [24]. Thus, these factors and processes will be able to determine changes in the concentration and bioactivity of anthocyanins, which will affect the degree of substance acceptance by the consumer [25]. In recent years, several studies have focused on the bioavailability of anthocyanins, proving that it would be less than 1%. At the same time, it was revealed that the bioavailability of anthocyanins depends on the glycosidic radical and, therefore, non-acylated anthocyanins are absorbed more efficiently than acylated ones [16]. Thereby, the stability of anthocyanins can be modified in three ways: by polymerization, cleavage and derivatization. The cleavage of anthocyanins results in colorless compounds, polymerization causes browning, and derivatization reactions cause the production of several molecules of different colors [26].

As such, in order to benefit from all the properties of the anthocyanins mentioned above, scientists have conducted numerous studies to understand how these parameters influence the stability and more precisely how to tackle these shortcomings to increase the stability and henceforth the effectiveness of anthocyanins.

The purpose of this research is to provide an overview of anthocyanin stability. In this respect, the most important factors that influence their stability were reviewed, and an analysis was performed on how anthocyanin degradation takes place under the action of these factors. Thus, each factor will undergo discussions throughout this paper, determining the positive or negative effects it produces on anthocyanin stability. For a better understanding of their activity, a series of figures and tables are also included. It is in this way that the opportunity for future studies is presented, towards improving their stability and their intensive use both in the food industry and medical field.

## 2. Antioxidant Potential of Anthocyanins

Reactive oxygen species (ROS) and reactive nitrogen species (RNS) are compounds of great importance for the immune system with a role in cell signaling and other body functions. However, the production of large quantities of these compounds, which the body can no longer eliminate with the help of endogenous and exogenous antioxidants, will cause alteration of the oxidative balance of the biological system, and the occurrence of oxidative stress [27,28,29]. Its appearance produces oxidative changes in molecules, tissue damage and causes accelerated cell death, all of which disrupt cell function, which is the first step in the emergence of many diseases [30].

A compound can be called an antioxidant if it delays or prevents the oxidation of a substrate at low concentrations. The basic property of an antioxidant is that it helps to limit oxidative damage in the human body by preventing or detecting a chain of oxidative propagation by stabilizing the produced radical. Substances with antioxidant capacity can act through multiple mechanisms such as: hydrogen atom transfer (HAT), single electron transfer (SET) or the ability to chelate transition metals [29].

The main endogenous antioxidants are enzymes such as superoxide dismutase, glutathione peroxidase and catalase, but certain non-enzymatic compounds may also have antioxidant functions, such as bilirubin, albumin, metallothionein and uric acid. In some cases, endogenous agents fail to provide effective protection and strict control against reactive oxygen species, so there is a need to administer exogenous antioxidants in the form of nutritional supplements or pharmaceutical compounds that contain an antioxidant. The most important exogenous antioxidants are vitamins C and E, certain minerals, β-carotene and flavonoids [31].

The most relevant compounds in the flavonoid class are anthocyanins, which have a flavylium cation structure, as is shown in the next chapter. Thus, most of the functional properties of anthocyanins, such as their sensory quality and antioxidant capacity are determined by their chemical structure, which acts as an acid. It is worth mentioning that the structure and properties of these polyphenols, including their antioxidant capacity, are influenced by certain factors such as temperature, pH and solvents, factors that should be controlled to obtain relevant results in terms of their antioxidant activity [32]. In addition, even the glycosylation of anthocyanins has been shown to decrease antioxidant activity and the ability to capture free radicals, compared to the aglycone form, thereby decreasing the power of anthocyanin radicals to delocalize electrons [27]. On the other hand, anthocyanin bioavailability influences the efficacy of antioxidant activity of this compound in oxidative stress [32]. In general, anthocyanins neutralize reactive radical species by transferring a single electron or by removing the hydrogen atom from phenolic groups. The central component of the antioxidant activity of anthocyanins is represented by the oxidation of phenolic hydroxyl groups, more precisely the para- and orthophenolic groups, which have a crucial role in the formation of semiquinones and in the stabilization of one-electron oxidation products [20].

Therefore, a large amount of anthocyanins are found in fruits and vegetables, so their consumption also involves the intake of a certain amount of antioxidants that will contribute to the protection of various types of diseases caused by oxidative stress [30]. However, we must remember that these foods include a variety of different phytochemicals and vitamins that may interact with anthocyanins in a synergistic or antagonistic manner, improving or lowering their antioxidant activity [27].

Undoubtedly, measuring the antioxidant activity of biological samples and food is of crucial importance, not only for ensuring food quality but also for determining the effectiveness of food antioxidants in preventing and treating diseases based on oxidative stress [33]. To this end, several antioxidant assays have been developed to evaluate anthocyanin capacity to inhibit the oxidation process that happens naturally [32].

Measurement methods for the antioxidant capacity of foods and various products have improved considerably in recent years, so it is necessary to establish and standardize measurement tests for the antioxidant capacity of various compounds [30]. In addition, evaluation methods for the antioxidant capacity must have certain characteristics such as speed and reproducibility and can be performed with small amounts of the analyzed compounds [29].

With these in mind, the most common methods for determining antioxidant capacity, which are able to assess this anthocyanin property, will be mentioned. There are two categories of such methods, one based on hydrogen atom transfer (HAT) and the second based on single electron transfer (SET). The methods based on the HAT mechanism include oxygen radical absorbance capacity (ORAC), while the types of assays that are SET based include: ferric ion reducing antioxidant power assay (FRAP), 2,2-Diphenyl-1-picrylhydrazyl radical scavenging assay (DPPH^•^) and cupric ions (Cu^2+^) reducing antioxidant power assay (CUPRAC). Likewise, specialist studies have reported that the method 2,2-Azinobis 3-ethylbenzthiazoline-6-sulfonic acid radical scavenging assay (ABTS^•+^) uses both mechanisms. At the same time, there are other types of tests that are not based on the aforementioned mechanisms that measure the sample scavenging ability for oxidants, which interact and produce a negative effect on major macromolecules in biological systems. One such test is hydrogen peroxide (H_2_O_2_) scavenging assay [30].

### 2.1. DPPH^•^ (Diphenyl-1-Picrylhydrazyl) Assay

DPPH^•^ is a common and frequently used spectrophotometric procedure for determining antioxidant capacities of components. This technique can be used on both solid and liquid samples, and is based on the ability of the free radical (DPPH^•^) to react with a hydrogen donor (AH^+^) [32]. Thus, electron donation by the antioxidant takes place to neutralize the DPPH^•^ radical [33].

This free radical is a stable one, but when the delocalization of the electron takes place, a purple color is obtained, which presents an intense abortion in the UV–vis spectral region at 517 nm [29,32]. When DPPH^•^ interacts with a hydrogen donor, the reduced form, DPPH, is formed; this results in the violet color vanishing. As a result, the decrease of DPPH^•^ gives an index for estimating the test compound capacity to capture radicals [29,31].

In conclusion, this technique is a simple, fast and economical one, used successfully to determine the antioxidant activity of some substances. In addition, the DPPH^•^ assay is the oldest indirect method of determining antioxidant activity and has been used for the first time in determining the antioxidant capacity of phenolic compounds [30].

### 2.2. Method of Inhibition of the (2,2′-Azinobis-(3-Ethylbenozothiazoline-6-Sulfonate)) Radical Cation (ABTS^•+^)

ABTS is also a simple spectrophotometric method, which measures the total antioxidant capacity [33]. This technique is initiated by the reaction of ABTS with persulfate potassium, which produces a stable cation radical (ABTS^•+^) of blue/green color that is absorbed at a maximum wavelength of 734 nm [29]; thus, absorbance decreases for this radical when it reacts with an antioxidant [32].

As part of this test, when ABTS interacts with the antioxidant, it forms ABTS^•+^, which has an intense color, and the antioxidant capacity of the substance is expressed as the ability of the test compounds to decrease the color that reacts directly with the ABTS radical [30]. As a result, the level of discoloration can be reported as a percentage of ABTS^•+^ inhibition, which is computed as a function of antioxidant concentration and time. ABTS assay is suitable for both lipophilic and hydrophilic compounds, can be used at various pH levels and is excellent for investigating the impact of pH on antioxidant activity [29,30].

At the same time, this method is important for determining the antioxidant activity of mixtures of substances, and has a role in distinguishing between additive and synergistic effects [32]. It should also be noted that the ABTS assay is frequently correlated with DPPH assay to determine antioxidant activity because both mechanisms of these methods involve the acceptance of electrons and H^•^ from antioxidant agents. This combination of tests is mainly used to determine anthocyanin antioxidant activity, and numerous studies have been conducted on various food matrices such as wine, pomegranate juice, blueberries and corn [32].

### 2.3. The FRAP (Ferric Reducing Antioxidant Power) Method

FRAP assay is a colorimetric method, which measures the ability of antioxidants to reduce the ferric ion complex (Fe^3+^) to the ferrous complex (Fe^2+^), which has an intense blue color [33]. Therefore, the increase of the absorption is measured at 593 nm and is related to a standard solution of ferrous ions or to a standard antioxidant solution to obtain the FRAP values [30].

Certain conditions are required in order to perform this test to determine the antioxidant capacity, namely, the FRAP test must take place in acidic conditions (pH = 3.6) to maintain the solubility of iron, the tested samples must be aqueous [32,33], and there must be an incubation time of 4 min at 37 °C to activate the antioxidant capacity of most samples [29].

In the past, the FRAP test used tripyridyltriazine (TPTZ) as an iron ion binding agent, but potassium ferricyanide is currently used. Thus, in the case of the use of potassium ferriyanide, Prussian blue is obtained as the final reaction compound that can be quantified spectrophotometrically and which shows the reducing power of the antioxidants that have been tested. Antioxidants can lead to the formation of Prussian blue by two different mechanisms. The first mechanism involves the reduction of Fe^3+^ from the solution to Fe^2+^ by binding ferricyanide, which will give the Prussian blue color, and the second mechanism is achieved by reducing ferricyanide to ferrocyanide that will then bind free Fe^3+^ [33].

The FRAP method is very easy, quick, reproducible and inexpensive, and it does not require any additional equipment. It has been used successfully on a large scale to determine the antioxidant activity of anthocyanins in various matrices, for example: in elderberry, raspberry, blackberry, red currant, carrot, cabbage, potato, onion and eggplant and even red wine [29,32].

### 2.4. Oxygen Radical Absorbance Capacity (ORAC) Method

ORAC assay is a fluorescence technique that involves the test sample (AH^+^), a fluorescent compound such as fluorescein or protein phycoerthrin (β-PE) and a free radical generator [32]. The most common free radical generators used for this test are azo compounds such as 2,2-azobis(2-amidinopropane) chlorhydrate (ABAP), 2,2′-azobis(2,4-dimethylvaleronytril) (AMVN), α,α,-azobisizobutyronytril (AIBN) and 2,2′-azobis(2-amidinopropane) dihydrochloride (AAPH) [33].

The mechanism of this method is based on the degradation of the fluorescent compound, as the interaction with free radicals determines its oxidation. However, when antioxidant agents (AH^+^) are present in the reaction, the fluorescent compound is protected from oxidative degradation, and thus, the fluorescence signal remains unaltered [32]. The ORAC test measures the capacity of antioxidants to produce hydrogen atoms, it assesses both the time effect and the degree of inhibition simultaneously [29], and hence, it is a HAT-based assay [33].

In the case of the ORAC test, certain reaction conditions are required for relevant results, the pH must be 7.4 and both fluorescein and the source of free radicals need to be kept at a constant temperature of 37 °C. The first stage of this procedure involves the thermal decomposition of AAPH—the free radical generator—which stimulates the degradation of the fluorescent substance. The introduction of the antioxidant agent, whose capacity is being tested, favors the elimination of peroxyl radicals, thus protecting the fluorescein from damage. The decrease of fluorescence is monitored at an interval of 1 min for 35 min at a wavelength of 485 nm for excitation and at 535 nm for emission. Finally, the change in fluorescence, due to the attack of free radicals but also the protection offered by antioxidants, generates a curve on the graph. Thus, the antioxidant capacity is determined from the area under the fluorescence decrease curve [29].

The use of the ORAC method is very frequent because it can be easily adapted to the type of sample, hydrophobic or hydrophilic, while solely the free radical generator needs to be changed [32]. Therefore, this assay has been widely used to assess the antioxidant content of drinks, supplements and fruits and vegetables [29]. Moreover, many authors have assessed the antioxidant activity of anthocyanins using this method [32].

### 2.5. CUPRAC (Cupric Ion Reducing Antioxidant Capacity) Assay

CUPRAC assay is similar to the FRAP method, a spectrophotometric technique, which measures the antioxidant capacity of a compound based on the reduction of the cupric ion (Cu^2+^) to cuprous (Cu^+^) [33]. The optimal pH for the CUPRAC test is 7.0, which is close to physiological pH (7.4) and mimics the antioxidant effect under real conditions [30].

Similar to the previously mentioned tests, this method also has a specific ligand, Neocuproine (2,9-dimethyl-1,10-phenanthroline), used to form a copper–ligand complex whose absorbance will be spectrophotometrically measured. In short, the reduction of Cu^2+^ to the Cu^+^ complex occurs in the presence of an antioxidant and neocuproine complex, which has a maximum absorption peak at 450 nm. Additionally, the reaction time required to complete the reaction depends on the speed of the antioxidant, thus varying between 30 and 60 min. It is to be mentioned that flavonoid glycosides may require preliminary hydrolysis to completely reveal their antioxidant potential [33].

This method is cost-effective, fast, stable and can be used on a wide range of antioxidant agents, regardless of the chemical or hydrophobic type. However, citric acid and simple sugars, which are not true antioxidants, cannot be oxidized by the CUPRAC reagent [30].

### 2.6. Hydrogen Peroxide (H_2_O_2_) Scavenging Assay

Hydrogen peroxide (H_2_O_2_) is a biological compound found in air, water, plants, microorganism and food at low concentration. H_2_O_2_ appears in the body as a by-product of normal aerobic metabolism. Thus, under stress, H_2_O_2_ is produced in excess and is harmful to cells because it could be transformed into ROS, such as hydroxyl radicals, which initiate lipid peroxidation and cause damage to DNA [34,35].

Furthermore, the H_2_O_2_ test detects reactive oxygen uptake and is an important aspect of total antioxidant activity. This assay evaluates the uptake capacity against H_2_O_2_ and relies on the principle of the intrinsic absorption of this molecule at a wavelength of 230 nm. For this method, a solution of H_2_O_2_ (40 mM) is prepared with phosphate buffer at a pH of 7.4. Therefore, after an incubation period of 10 min, the remaining concentration of H_2_O_2_ is measured spectrophotometrically and compared to a control solution that includes phosphate buffer without H_2_O_2_. The absorbance value at 230 nm falls when the H_2_O_2_ concentration decreases in the presence of scavenger agents [30].

Thus, a colorimetric test was created in order to evaluate the capacity of capturing the H_2_O_2_ by antioxidants, especially by plant extracts. This assay is based on the reaction between H_2_O_2_, phenol and 4-aminoantipyrine in the presence of horseradish peroxidase (HRP) and produces a pink compound, namely quinonimine. As such, antioxidant agents will decrease chromatophore production, changing the color of the solution [36].

In conclusion, this approach is used to test the hydrogen peroxide scavenging capacities of common antioxidants such as ascorbic acid, gallic acid and tannic acid, as well as selected plant extracts [36].

Considering all these different evaluation methods for antioxidant activity, Table 1 includes the main advantages and disadvantages of each method.

**Figure 3:**
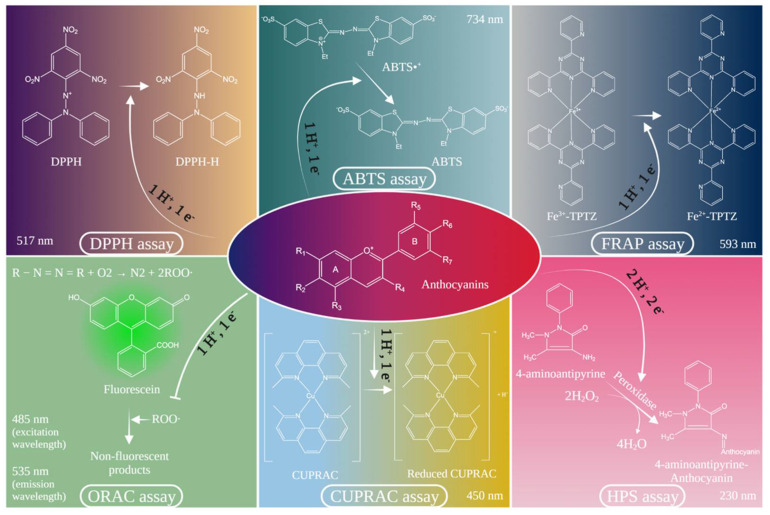
The action mechanism of methods for evaluating antioxidant activity.

In addition, for a better understanding of these methods to determine the antioxidant activity of phenolic compounds, specifically anthocyanins, Figure 3 includes all the presented tests, compounds used and their mechanism of action.

Due to the very high antioxidant potential of anthocyanins, but also the other health benefits, it is necessary to study the factors that influence their bioavailability and implicitly the antioxidant capacity. To this end, the most important parameters that affect the bioavailability of these polyphenols will be highlighted.

## 3. The Influence of pH

**Figure 4:**
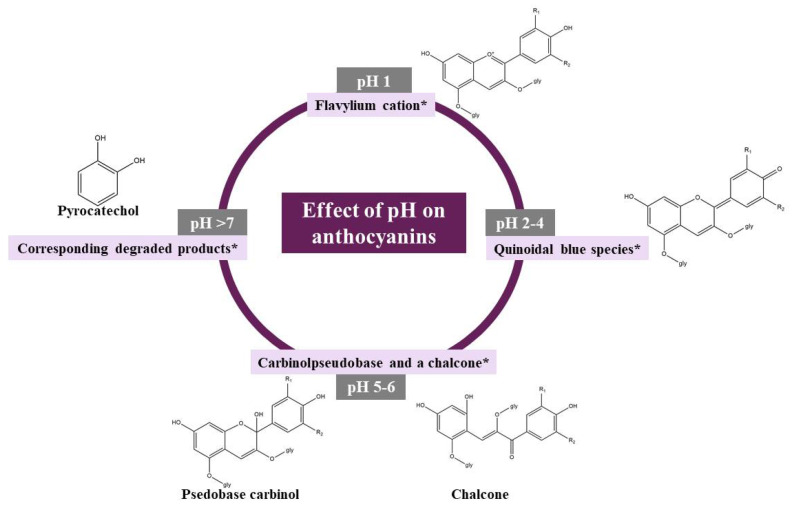
Anthocyanins at different pH values, * predominant chemical form [42].

Due to the ionic nature of the molecular structure, the color of anthocyanins is influenced by pH, as this is the first parameter that is approached. As it is known, anthocyanins are found in four different chemical forms, which depend on the pH of the solution. Thus, in an acidic environment, at pH = 1, anthocyanins are found in the form of the flavylium cation (red color) which makes them very soluble in water [12], and this form is also responsible for the production of red and purple colors. When the pH increases between 2–4, the quinoidal blue species is found abundantly, while at a pH between 5–6, carbinol pseudobase and a chalcone appear, compounds that are colorless [38]. Finally, at a pH higher than 7, the anthocyanins will be degraded according to the substituent groups. However, the four forms of anthocyanins can co-exist at a pH range of 4–6, the balance of these forms being maintained by the flavylium cation (Figure 4) [39,40]. Generally speaking, ring B substituents and the presence of additional hydroxyl or methoxyl groups are responsible for the stability of anthocyanins, which in neutral environments decrease aglycone stability. On the other hand, although in a neutral environment, aglycones are not stable, monoglycosides and diglycoside derivatives are more stable under these conditions, because sugar molecules will avoid the degradation of unstable intermediates into phenol and aldehyde acid molecules [15,26]. In short, the stability of anthocyanins increases with increasing methylation and decreases with an increasing number of hydroxyl groups in the B ring of anthocyanidins. At the same time acylation plays an important role in improving the stability of anthocyanins [41].

## 4. Co-Pigmentation Effect

The following paragraph will focus on another factor that influences the stability of anthocyanins, namely co-pigmentation, this phenomenon is specific to anthocyanins, and cannot be observed in other classes of polyphenols or other non-phenolic compounds [43].

Co-pigmentation is the process by which pigments form molecular or complex associations with other colorless organic compounds or metal ions, thus producing a change or increase in color intensity [44]. For a molecule to act as a co-pigment, it must meet two requirements: to possess sufficiently extended π-conjugated systems, which are supposed to favor π−π stacking interaction, and to have hydrogen bond donor/acceptor groups, such as OH and C=O groups [45]. Thus, the solution of anthocyanins with the co-pigment will have a much more intense color than would theoretically be expected at the pH value of the medium [46]. It should be mentioned that this co-pigmentation phenomenon is pH dependent, because an increase in the pH value will cause the destruction of anthocyanins and, therefore, a decrease in the color intensity of the solution [47]. At the same time, the stability of co-pigmented complexes is influenced by thermal degradation. Therefore, at high temperatures the co-pigmentation mechanism has no visible effects or can even lead to opposite results. For example, co-pigmentation with ferulic acid, sinapic acid or heat treatment at 90 °C for 12 h or 88 °C for 2 min resulted in accelerated degradation of anthocyanins [48]. In addition, the interaction between anthocyanins and co-pigments is different depending on their nature and concentration, thus obtaining different shades and intensities of color [46]. The co-pigments are colorless in a free form, but when mixed with anthocyanins there is an interaction, which results in a hyperchromic effect and a bathochromic shift change in the absorption spectra, as pigment-free anthocyanins absorb light at a certain wavelength, and after co-pigmentation this value increases significantly. As such, the co-pigment anthocyanin complex will have a longer absorption wavelength [49,50]. The nature of these compounds can be extremely varied, therefore, pigments can be alkaloids, amino acids, nucleotides, organic acids, flavonoids, metals or even other anthocyanins [39,51]. The efficiency of the co-pigmentation effect depends on the steric arrangement of the substance (the best co-pigmentation effect occurs in the case of a planar structure) and its size, in order to achieve the stabilization of the flavylium ion [46].

Studies reveal that the main mechanism used to stabilize the color of anthocyanins is co-pigmentation with other substances. This phenomenon is possible due to the electron-rich structure of the co-pigments, so it is associated with flavylium cations that are poor in electrons, thus producing stabilization. Furthermore, this association protects the flavylium ion from the nucleophilic attack of water by generating steric hindrance [45]. The interaction between anthocyanins and co-pigments can be achieved in several ways, depending on the nature of the co-pigment. As such, an association between anthocyanins and colorless co-pigments is called inter-molecular co-pigmentation. An intra-molecular association appears when an interaction exists between the chromophore and nonchromophoric parts of the same molecule. There is also an inter-molecular interaction between anthocyanidin nuclei or between anthocyanidin nuclei and aromatic acyl groups called self-association [10]. If the co-pigment is metallic, a complexation occurs, and there are also more complex cases of co-pigmentation when anthocyanins form compounds at the same time with several molecules such as aglycones, sugars, co-pigments and protons. A special case of co-pigmentation occurs when anthocyanins form an interaction with another phenolic compound, this interaction is transitory due to the lack of chemical bonds. This type of bond is the result of chemical phenomena known as charge–transfer complex formation or π-π interactions and occurs when the interacting compounds are electrically charged differently. Therefore, in our case, because the flavylium ion has a positive charge, it is a good candidate for the formation of complexes by electronic charge transfer with rich electronic substrates [39,45].

### 4.1. Metallic Interaction

A type of metal co-pigmentation is used in the food industry, but there is an increased risk of contaminating products with metals, leading to their becoming toxic to consumers. However, the use of metals to stabilize the color of anthocyanins could be a successful method as long as the metals used are not a risk to the health of the individual and they are essential minerals in a balanced diet [39]. Thus, in this type of co-pigmentation, the metal cation has the ability to modify the absorption spectrum of the pyrylium ring by affecting the distribution of unlocated electrons. The strongest color effects can be observed when co-pigmentation is conducted with positively charged alkaline earth metals or with poor metals (+2, +3) [13]. Among anthocyanins, the only compounds derived from cyanidin, delphinidin and petunidin are capable of metal chelation, due to the free hydroxyl groups in the B ring. The most common metals that can form complexes with anthocyanins are copper (Cu), iron (Fe), magnesium (Mg), tin (Sn) and potassium (K) [45,52]. In addition, some authors believe that the blue color of plants is the effect of the interaction of anthocyanins and metals. For example, the interaction between anthocyanins and molybdenum is thought to be responsible for the stabilization of the blue color in Hindu cabbage tissue [42]. Current research has found that the interaction between o-di-hydroxyl anthocyanins and Fe (III) or Mg (II) ions placed in a solution with pH = 5 is responsible for the formation of blue color in plants, which is possible if the stoichiometric ratio between anthocyanin and Fe (III) is 1:6 [39,53].

Finally, this process can reduce the concentration of free ions in the cell, thus minimizing the possible harmful effects of metal toxicity on plants. According to research, it has been shown that toxic metals that form complexes with anthocyanins will be sequestered in vacuoles, thus the ions of these metals are accumulated in non-photosynthetic tissues, which are less sensitive and, therefore, important processes in plant growth and development will not be affected. At the same time, they play a role in the correct assimilation of carbon. These properties of anthocyanins have important biotechnological implications as plants that have a large amount of anthocyanins can be used to phyto-remedy soils contaminated with toxic metals [54].

### 4.2. Self-Association

Anthocyanins can form bonds with other anthocyanins, this type of interaction being called self-association. The obtained complex does not present a degree of association as strong as co-pigmentation, as a higher concentration of anthocyanins is necessary in order to produce and observe this phenomenon. However, there are some stronger self-association interactions than others in the case of neutral species, but they can be destabilized due to the rejection between positively charged flavylium cations and negatively charged anionic bases [55]. In addition, it has been observed that anthocyanin molecules are arranged vertically in chiral (helical) aggregates, with left geometry. Furthermore, an important role in strengthening the structure is played by the hydrogen bonds between the sugars attached to the anthocyanins; however, the position and size of the sugar are responsible for the overall alignment of the complex [46,55]. This process has been observed during the aging of wine and is supposed to play a role in determining the color of aged red wines. However, future research is necessary to discover the mechanism responsible for this process [52].

### 4.3. Intramolecular and Intermolecular Co-Pigmentation

**Figure 5:**
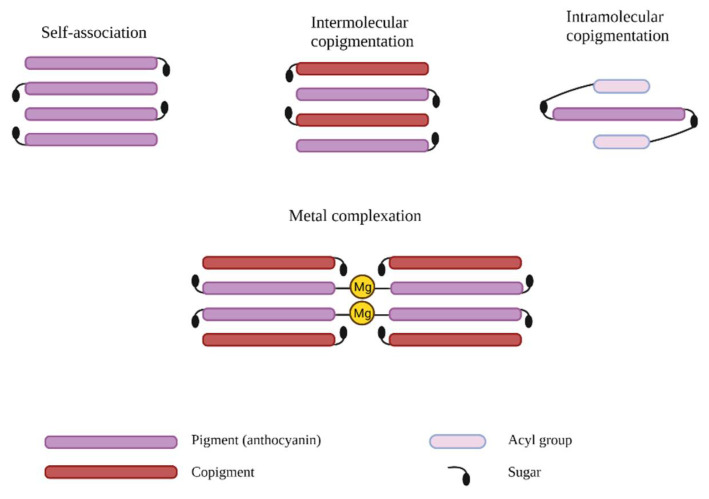
Types of anthocyanin co-pigmentation.

Intramolecular interactions are performed by stacking the hydrophobic acyl moiety covalently bound to sugar and the flavylium nucleus, thus reducing the hydrolysis of anthocyanins [56], and intermolecular interactions occur due to van der Waals forces between the planar polarizable nucleus of the anthocyanin and colorless co-pigment (Figure 5) [57]. Intermolecular co-pigmentation frequently occurs in red wines, due to the already mentioned van der Waals interactions, but also due to hydrophobic effects and hydrogen bonds, which achieve a non-covalent interaction between anthocyanin molecules and other molecules (co-pigment). Thus, these interactions impart the wines with more shades of purple and a darker color, through the two occurring actions, namely, the hyperchromic effect and the bathochromic one. At the same time, it is assumed that this color change is the result of changing the conformation of the anthocyanin chromatophore, but also avoiding the hydration reaction, thus determining an increase in the proportion between the flavylium cation (red color) and the quinonoidal base (purple color) [58].

This interaction plays an important role in preventing whitening during light and heat treatments, which is necessary to obtain certain products [59]. Additionally, inter-molecular co-pigmentation plays an important role in the case of anthocyanin–metal complexes that we have already discussed and which are a special case [49], but unlike metal complexation, intermolecular interaction occurs in all major anthocyanidins [52].

Finally, intermolecular co-pigmentation took place in vacuolar fluids but also in extra vacuolar ones, and this is the main mechanism responsible for the multitude of colors that appear in flowers, considering the limited number of existing anthocyanins [46].

On the other hand, intra-molecular co-pigmentation requires an interaction between the anthocyanidin backbone and a co-pigment forming part of the anthocyanin itself. This phenomenon occurs due to the alignment of the aromatic acyl fragments of the acylated anthocyanins and the anthocyanidin core structure, the structural characteristics of anthocyanins are the main determinants of the effectiveness of this interaction. In addition, the sugar from the anthocyanin structure acts as a spacer between the anthocyanin skeleton and the acyl moiety, thus allowing the molecule to fold in a way that the aromatic acyl group will interact with the π-system of the planar pyrylium ring protecting it against the nucleophilic water attack [43,46]. Due to the formation of sandwich structures (the anthocyanin core will be incorporated between two acyl portions), an efficient stabilization of anthocyanins pigments will be achieved for those having two or more aromatic acyl portions necessary to achieve this complex [43].

As anthocyanins from flowers are most often found in acylate form, intramolecular interactions occur that stabilize the color of the flowers even at neutral pH values. This phenomenon takes place not only in flowers but also in the case of other edible plants that have a high content of acylated anthocyanins, for example, black carrot [52]. Thus, it is assumed that due to the bonds formed in intramolecular co-pigmentation, this interaction is more efficient and strong than intermolecular co-pigmentation in stabilizing the color of anthocyanins [52]. Finally, due to the molecular interactions between anthocyanins and other uncolored phenolic components, it has been shown that all types of co-pigmentation reactions have a beneficial effect on improving the stability of anthocyanins [50].

## 5. Temperature

As is well known, heat processing is one of the most commonly used methods for preserving and extending the shelf life of food and ensuring food safety. Thus, depending on the functional parameters of the food and the shelf life desired by the manufacturer, heat treatment can take place at temperatures between 50–180 °C. Due to this processing under high temperature for certain periods of time, foods can undergo changes in color, the amount of anthocyanins but also their antioxidant capacity [25]. Due to heat processing, anthocyanins can undergo a multitude of mechanisms such as glycosylation, nucleophilic attack of water, cleavage and polymerization that will cause the loss of this pigment and their degradation [57]. Therefore, temperature is another factor that affects the stability of the molecular structure of anthocyanins, so with increasing temperature the degradation of these compounds occurs [60] and determines the browning of products in the presence of oxygen [42]. As such, both the color intensity determined by monomeric anthocyanins and their amount decreases depending on time/temperature, while the amount of brown pigments/polymer fraction increased. It has been proven that in all food pigments, including anthocyanins, stability decreases with increasing temperature. At the same time, current research has reported that the stability of these compounds is closely related to their chemical structure, where the sugar fraction is an important factor [61].

According to a study by Turker and colleagues in 2004, anthocyanins stored at different temperatures, in an acylated form, have greater stability than nonacylated anthocyanins [62]. Another study that analyses the influence of storage temperature on anthocyanin content and their half-life was performed by Hellström and colleagues in 2013. The current study shows how storage temperature affects the stability of anthocyanins, one of the most important quality criteria in berry juices. Therefore, the half-life (t½) of anthocyanins was considerably shorter at room temperature than in cold storage in all juices examined. In this respect, in the long-term efficient conservation of anthocyanins, it is recommended to avoid storing them at room temperature [63].

On the other hand, research on grape extracts reported that heat treatment at 35 °C reduced the total anthocyanin content to less than half, compared to the same grape extract that was subjected to a heat treatment of only 25 °C. In addition, the color of anthocyanins changed from red to orange up to temperatures of 40 °C, a phenomenon observed in an environment with acidic pH, which did not influence this change [64].

Thermal processes that require high temperatures such as bleaching and pasteurization at 95 °C degrees Celsius for 3 min to obtain blueberry puree, caused a loss of 43% of total monomeric anthocyanins, compared to the amount observed in fresh fruit before heat treatment. It should be noted that while the amount of anthocyanins decreased, the values of polymer colors increased from 1% to 12%. This finding implies that heat labile parameters can promote anthocyanin pigment degradation, and it backs up the theory that pigment destruction in juice processing is caused by endogenous enzymes in fruits [65]. Although, so far, it has been shown that heat treatment has a negative effect on the amount of anthocyanins in fruits and vegetables, it can still have a beneficial effect. Polyphenols, so implicitly anthocyanins, are enzymatically degraded by polyphenol oxidase, but this enzyme can be thermally inactivated [65,66]. As a consequence, in the food processing sector, mild heat treatment of raw materials, such as blanching, can reduce anthocyanin oxidation by polyphenol oxidase [12]. Therefore, a short-term heat treatment has been shown to improve the stability of anthocyanins by inhibiting native enzymes that are harmful to anthocyanins [66,67]. At the same time, thermal degradation can be prevented by decreasing the pH value of the anthocyanin solution. Furthermore, the decrease in oxygen concentration, a parameter that will be discussed further, has been shown to protect anthocyanins from thermal degradation [52,68].

## 6. Oxygen

Due to the unsaturated chemical structure of anthocyanins, these compounds are susceptible to reaction with molecular oxygen [69]. Therefore, oxygen is another important factor that influences the stability of anthocyanins, having a role in their degradation, as the presence of oxygen can accelerate the degradation process of anthocyanins in two ways: by a direct oxidative mechanism or by the action of oxidizing enzymes [65]. This factor causes a detrimental effect on anthocyanins and numerous studies have reported that the stability of these compounds increases when stored under vacuum, nitrogen or argon, compared to their storage in an atmosphere containing predominantly oxygen. Therefore, the concentration of anthocyanin dropped in all of the atmospheres studied; where high oxygen produced a greater decline [61]. As mentioned in the previous chapter, removing oxygen from the anthocyanin solution prevents thermal degradation. In fact, high temperature and the presence of oxygen have proven to be the most harmful combination of all the factors that influence the stability of these compounds [70]. For many years, it has been known that filling bottles completely with hot grape juice may postpone the color degeneration from purple to dull brown. Other juices containing anthocyanins have yielded similar results [69]. In addition, because anthocyanins react with oxygen radicals, such as peroxyradicals, they have an antioxidant character, rendering these compounds beneficial against cardiovascular diseases [52,71].

On the other hand, some investigators concluded that if food is stored in an environment enriched with 60–100% oxygen and at a low temperature, during the onset (0–7 days) of cold storage, the content of phenols and anthocyanins will increase. This effect, however, reduces with time spent in storage [70].

## 7. Ascorbic Acid

Ascorbic acid is recognized as performing a significant role as an antioxidant in the human body [72]. This compound is crucial in food processing because any change in vitamin C level shows that the quality of the food has deteriorated after processing and during storage [73]. Ascorbic acid is found in various fruits and vegetables, and, in addition, it is added as an antioxidant in many types of foods to increase their nutritional value. It is also known that the presence of ascorbic acid in an environment containing anthocyanins causes their faster degradation and loss of color, thus suggesting a direct interaction between the two molecules [69,74]. At the same time, the presence of oxygen in the environment will favor faster degradation of anthocyanins by ascorbic acid, thus determining the formation of the polymeric pigment and the whitening of the anthocyanin pigment. The specific process of degradation is yet unknown; however, adding ascorbic acid to anthocyanins increases the rate of decomposition of both molecules. The presumed mechanisms by which this phenomenon would occur are the direct condensation of ascorbic acid with anthocyanins, or the formation of hydrogen peroxide and the oxidative cleavage of the pyrylium ring by peroxides [75]. The loss of anthocyanin pigments by the second mechanism, namely the oxidative cleavage of the pyrylium ring, is due to the ability of anthocyanins to act as a molecular oxygen activator, the result of this reaction being free radicals [76].

On the other hand, electrophilic molecules, such as bisulphites, hydrogen peroxide, and ascorbic acid are believed to target anthocyanin nucleophilic sites. It has been proposed that ascorbic acid causes reciprocal and irreversible degradation of both pigment and micronutrients. This process is distinct from bisulfited bleaching, which is reversible and pH sensitive. Therefore, this phenomenon is a significant barrier to the use of anthocyanins-based colorants in the food sector, particularly in juices and drinks that are frequently fortified with vitamin C [74]. However, the effect of ascorbic acid on anthocyanins is a complex one, depending on several factors but also on the matrix [67]. For example, in an environment with H_2_O_2_, concentrations between 60–80 mg/L of ascorbic acid favored the stabilization of anthocyanins in pomegranate juice, while in cherry juice a concentration of 80 mg/L produced rapid degradation of anthocyanins [77]. Therefore, when both oxygen and ascorbic acid levels are high, the degrading impact is most evident. Copper ions are known to speed up the processes [61].

## 8. Light

Plants are exposed to light, which stimulates anthocyanin synthesis and accumulation, hence, light is another important parameter in the stability of these compounds [69]. However, anthocyanins are affected by light in two ways. Light is required for anthocyanins production, but it also speeds up their breakdown [70]. In addition, the amount of molecular oxygen present affects the rate of light-induced breakdown. It should be mentioned that when pigments are exposed to fluorescent light, the most intense anthocyanin loss occurs [61]. These compounds are responsible for determining the colors orange, red and blue in many plants, such as grapes and berries, as anthocyanins are good absorbers of visible light. Therefore, the color is mainly influenced by the aglycon’s B-ring substitution pattern, as opposed to the flavan structure glycosylation pattern, which impacts color production to a smaller extent [61]. Over the years, studies have been conducted on the impact of light on the stability of anthocyanin extracts from various biological sources. Thus, in the case of grape juice that was stored at a 20 °C temperature in the dark, it was observed that about 30% of the anthocyanin amount was destroyed. Nevertheless, placing the identical samples in the presence of light at the same temperature and duration removed nearly half of the total pigments [78].

Light, in addition to the above-mentioned information, also affects the antioxidant activity of anthocyanins. Thus, a significant decrease in anthocyanin content and antioxidant activity was observed in mulberry fruit extracts that were stored at room temperature and exposed to fluorescent light for 10 h. Thus, the longer the exposure of the extract to light, the more the anthocyanin content and their antioxidant activity will decrease [79].

In order to minimize the negative impact of light on anthocyanins, packaging can be made of materials that can block light from the visible spectrum and especially from the ultraviolet field of the spectrum, thus creating a protective barrier. In addition, glycosylation, acylation and co-pigmentation play an important role in increasing the stability of anthocyanins to light [61].

## 9. Sulfites

Although widely used in the storage of fruit derived foods, sulfites and sulfates can cause the loss of anthocyanin pigment. This discoloration is caused by forming colorless sulfur derivative structures when these sulfur compounds are added in positions 2 or 4 [70]. A quantity of sulfur dioxide will be released from the anthocyanins when heated, and it is in this way that the specific color of these compounds can be partially recovered. Acidification to a low pH also regenerates anthocyanins by releasing SO_2_. For instance, sulfur dioxide is widely used in the fruit and vegetable preservation industry, as an inhibitor of microbial growth and enzymatic and non-enzymatic browning [61]. On the other hand, high sulfite concentrations (more than 10 g/kg) trigger permanent anthocyanin degradation [70].

Sulfur dioxide has been used for food preservation since the 18th century, and has been shown to be an excellent choice for low-pH foods such as wine [80]. Therefore, SO_2_ is intensely used in the wine industry, due to its antiseptic properties against yeasts and bacteria and its antioxidant properties, making it one of the most versatile and efficient additives [81,82]. Its molecular form may cross the cell membrane of bacteria, disrupting the action of cell enzymes and proteins, and thus regulating microbial development [80].

These properties of sulfur dioxide play a role in stopping unwanted fermentations, such as malolactic and acetic fermentation, and limit the activity of oxidase that is endogenous in grapes or may come from fungal infection. In addition, SO_2_ causes pigment bleaching and eliminates unpleasant odors that are obtained from oxidation [81].

Furthermore, it acts as a solvent during winemaking, allowing grape solid components such as stems, seeds and skins, to be extracted. Additionally, SO_2_ is utilized in another three stages of wine making. First, it is used during the prefermentation process, in the grapes or must, with the primary goal of avoiding oxidation. However, this phenomenon does not occur by direct elimination of oxygen from edibles, but by binding to the precursors involved in oxidative reactions and compounds resulting from oxidation [80,82]. Second, after the fermentation procedures are ended and before the aging or storage phases, it is used to limit microbial development that might affect the wines, and last, shortly before bottling, the wines are stabilized with SO_2_ to avoid any changes or accidents in the bottles [82].

**Figure 6:**
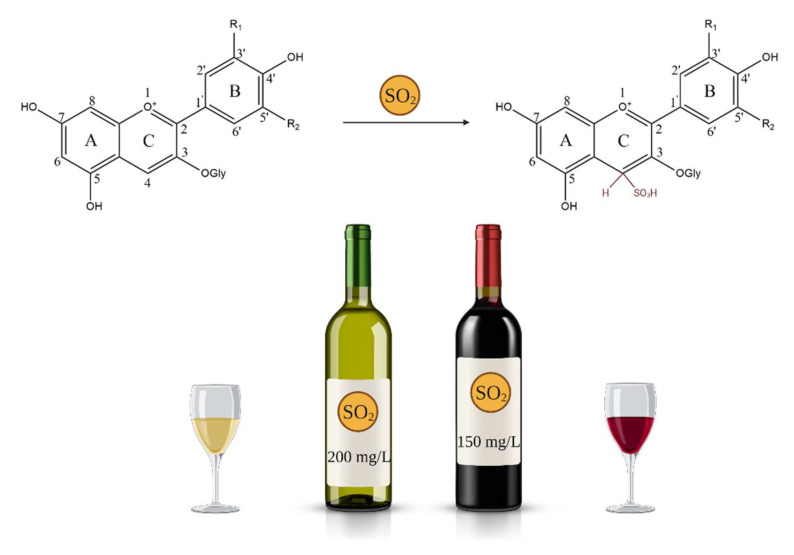
Content of SO_2_ in red and white wines, and anthocyanin reaction with SO_2._

Therefore, it can be said that this treatment with sulphur dioxide is one of great importance in wine technology. However, the use of this agent is strictly controlled, because high doses of SO_2_ can cause organoleptic changes of the final product (such as unwanted flavors in sulphur gas or reducing products, hydrosulphate and mercaptans). At the same time, the ingestion of this compound produces negative effects on human health [82]. In a number of individuals, it causes a variety of symptoms associated with allergic responses. As a result, the maximum SO_2_ content in wines authorized by law has gradually been decreased to 150 and 200 mg/L for red and white wines, respectively. Thus, at present minimizing or eliminating the use of SO_2_ in vinification is being sought, through research on innovative technologies (Figure 6) [80].

## 10. Enzymes

The most common enzymes that degrade anthocyanins are glycosidases, peroxidases (phenol oxidases) and phenolases (polyphenol oxidases) [42]. The collective name for these enzymes is anthocyanases [69]. These enzymes may be produced by the plant and are present in its tissues, or may occur as an effect of microbial contamination. It is worth noting that glycosidases directly affect anthocyanins, while peroxidases and phenolases have indirect effects on their stability [83].

Therefore, glycosidases break the covalent bond between the glycosyl residue and the aglycone of an anthocyanin, and thus unstable anthocyanidin are formed [42]. This process further affects the color of the compound [83]. On the one hand, polyphenol oxidase and peroxidase, which are plant-specific enzymes, once removed from cellular compartments during anthocyanin extraction, will accelerate the degradation of these compounds [67]. On the other hand, polyphenol oxidase is the enzyme that will catalyze two types of reactions: o-hydroxylation of monophenols in o-diphenols and oxidation of o-diphenols in o-quinones in the presence of molecular oxygen. Quinones are highly reactive electrophilic compounds that can covalently alter nucleophiles like anthocyanins to produce brown pigments known as melanin. Although anthocyanins are weak substrates for this enzyme, polyphenol oxidase reacts directly with the compounds, causing their degradation by a cooxidation reagent of enzymatically generated oquinones and/or secondary oxidation products of quinone [66]. It is assumed that the first enzyme that affects the stability of anthocyanins is β-glucosidase, which forms anthocyanidins that can be further oxidized by polyphenol oxidase and/or peroxidase [83]. Following the action of these enzymes, the solubility of anthocyanins decreases and their transformation into colorless compounds takes place, thus losing the color intensity of these pigments [69].

## 11. Encapsulation of Anthocyanins

The first step required before encapsulating anthocyanins in various systems is their extraction from natural sources. Thus, solvents that do not interfere with physical and antioxidant properties of anthocyanins are used. For the food applications of these compounds small organic alcohols are recommended, because they can efficiently permeate plant tissue, exert great affinity, and have a high solubility for anthocyanins. The most used and efficient solvents for the extraction of anthocyanins are acidified organic solvents, such as water, acetone, ethanol and methanol. Their acidification is achieved with weaker organic acids, such as acetic acid, but hydrochloric acid can also be used, with the role of improving the stability of anthocyanins [84].

As already mentioned, anthocyanins have multiple health benefits, but their effectiveness is limited by their low stability, which is influenced by the factors previously described such as pH, temperature, light, oxygen and others. Furthermore, due to their low absorption and rapid metabolization in the organism, these compounds have very low bioavailability and the best strategy to overcome these disadvantages is to create anthocyanin-encapsulation systems [85,86].

Encapsulation can be defined as the process by which a bioactive agent of nature: solid, liquid or gaseous is covered by a polymeric material or introduced into a matrix in order to protect it from harmful environmental factors. Through this process, certain parameters can be controlled such as place and time of delivery for maximum efficiency [85,87]. The size of vesicles produced by this method varies, ranging from 1 to 1000 μm [88].

**Figure 7:**
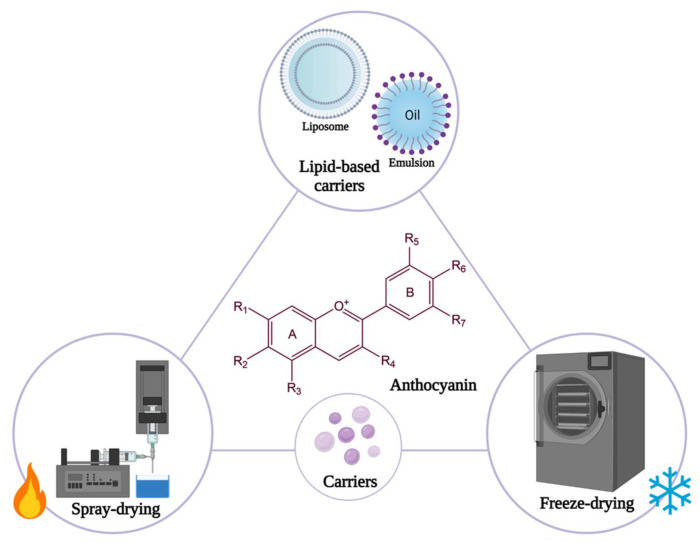
Methods for anthocyanins encapsulation.

In recent years, a great number of techniques for anthocyanin encapsulation have been developed and optimized in micro- and nanocarriers. There are several methods of obtaining compounds that contain anthocyanins, the most common are spray-drying, freeze-drying or electro-spinning/spraying. At the same time, lipid-based carriers can be obtained for anthocyanins, such as liposomes and emulsions [85]. Some of the following methods of encapsulating anthocyanins and their characteristics are of paramount importance and are presented in Figure 7.

### 11.1. Spray-Drying

According to studies, spray-drying is the oldest and the most frequent method used for microencapsulating anthocyanins. For the encapsulation of anthocyanins by this technique, certain steps are necessary: first the fluid feed is atomized through a high-pressure nozzle into a drying chamber, into a solution, emulsion or suspension. The next step involves the evaporation of the solvent used by heating the environment where the sprayed drops are found to 150–220°. As the final step, a filter or cyclone is used to separate and recover the powdered product from the air [85,88].

This method is quick, adaptable, cost-effective and simple to scale up, with high encapsulation efficiency and relatively good storage stability [88]. In addition, various types of encapsulating agents can be used such as lipids, proteins and polysaccharides depending on the type of incorporated compound [89]. A crucial role for the efficiency of encapsulating compounds by this method, is the choice of a suitable wall material for microencapsulation by spray drying. However, the range of compounds that can be chosen for this technique is limited, because the agent must meet certain criteria such as: low viscosity at high concentrations, acceptable solubility, film-forming capacity and emulsifying properties [88]. Bearing these criteria in mind, the most used compounds are polysaccharides for spray-drying encapsulation of anthocyanins, because they have low viscosity, desirable solubility and adequate emulsification characteristics, but also high capacity to retain volatile compounds. Therefore, they are widely used in microencapsulation of polyphenolic compounds [85].

Following this technique, non-uniform particles of various sizes are obtained, so in spray-drying the size and morphology of the carries cannot be controlled. To this drawback is added the fact that the high temperatures used in the process can degrade anthocyanins [88].

### 11.2. Freeze-Drying

This technique uses an opposite procedure to spray-drying, using low temperatures for dehydration. Therefore, freeze-drying, also known as lyophilization due to the low temperatures used, is suitable for encapsulating high temperature sensitive compounds, such as anthocyanins. [88,89]. The steps of this technique include freezing, sublimation (primary drying), desorption (secondary drying) and finally the storage of the resulting dry material [90].

The advantages of freeze-drying rely on the fact that it is a simple process, which takes place in the absence of air and at a low temperature and, as a result, the obtained compounds are resistant to oxidation or chemical modification [90]. This technique is used to improve thermal and color stability especially for anthocyanins, using different wall materials [89]. However, the freeze-drying method also has disadvantages such as high costs due to the vacuum technology needed, and a long period of time for dehydration, about 20 h [89,90].

### 11.3. Liposomes

Liposomes are phospholipid vesicles of a spherical shape composed of one or more concentric lipid bilayers comprising an aqueous space [86]. In recent years, methods and systems for obtaining liposomes have advanced considerably, but there are several basic steps in this process. The first step is lipid drying from organic solvents, then dispersion of lipids in an aqueous medium. Afterwards, liposomes need purification, and are followed by post-processing steps such as sonication or extrusion [91]. Thus, depending on the method of production, the compounds used and the environmental conditions, liposomes can have different sizes and structures [90].

Due to their good ability to encapsulate and protect hydrophilic substances, liposomes are used to encapsulate anthocyanins. Therefore, by incorporating these polyphenols, they are protected from degrading environmental factors. At the same time, liposomes can increase absorption and bioavailability of anthocyanins due to their biocompatibility, amphiphilicity, nontoxicity and non-immunogenicity, [88,89]. All these advantages have contributed to the growing interest in these vesicles, having versatile applications in the biomedical, food and cosmetic industries [89].

Despite the many advantages presented, they can undergo oxidation processes due to unsaturated fatty acids in the membrane component, leading to the formation of hydroperoxides. The high costs of manufacturing and the fact that there is no standardization of methods of production, require future research and add to the estimated shortcomings [88,89].

### 11.4. Emulsion

Emulsions consist of at least two immiscible liquids, most commonly water and oil, and surfactants, in which one of the liquids is dispersed in small spherical droplets into the other. They can be found in different forms depending on the liquid that is dispersed, so we have simple emulsions such as oil in water (O/W) or water in oil (W/O), and double emulsions, oil in water in oil (O/W/O) or water in oil in water (W/O/W) [85,89].

Likewise, a double emulsion of water in oil in water (W/O/W) is a suitable method for the encapsulation of hydrophilic substances, such as anthocyanins [88]. As such, anthocyanins can easily be incorporated into the dispersed phase of an emulsion, thus protecting them from harmful environmental factors and against degradation. At the same time, this encapsulation system controls the release of anthocyanins at a certain time or place and in addition improves their bioavailability in the gastrointestinal tract [89].

Although they manage to effectively encapsulate anthocyanins, the main disadvantage of emulsions is that they are thermodynamically unstable systems, and tend to break down with time [89].

## 12. Conclusions and Future Perspectives

The aim of the present review has been to gather the scientific information of the antioxidant capacity of anthocyanins and its evaluation methods, and the main parameters that influence anthocyanin stability and encapsulation methods. There is a wide variety of factors that can affect their stability and, implicitly, their bioavailability. Since anthocyanins are the most common and widely used category of water-soluble natural colors, a large number of studies have been conducted to improve their stability, in order for them to be used in different fields, from the food industry to the cosmetics industry. Their main use is as food dyes, as they impart a wide range of colors and can replace widely-used synthetic dyes nowadays. The latter can exhibit toxicity and adverse health effects. In addition to their main use, as pigments, anthocyanins also have important antioxidant, anticancer, antitumor, antimutagenic and antidiabetic characteristics. To get the most out of these anthocyanin properties, it is important to know how their stability is influenced by pH, temperature, co-pigmentation, oxygen, ascorbic acid, light, sulfites or enzymes.

In order to extend their efficiency, new directions of research involve the incorporation of anthocyanins into targeted delivery systems, and, therefore, the load of the compound is projected by the external environment that could influence their stability. Thus, various systems such as liposomes, microcapsules or emulsions have been developed to increase their bioavailability, so, implicitly the effects produced by anthocyanins would be stronger. However, future studies are needed to focus on the action of each factor that influences stability and how to reduce the negative impact they have on it.

## Figures and Tables

**Table 1:** Common assay for antioxidant activity, their reaction, advantages and disadvantages.

Method	Reaction	Advantages	Disadvantages	References
DPPH^•^ assay	ArOH + DPPH^•^ →ArOH^•^ + DPPH_2_	- can be used to test hydrophilic and lipophilic antioxidants with polar and nonpolar organic solvents	- can interact with other radicals, - its stability is sensitive to certain solvents	[29,37]
ABTS^•+^ assay	(NH_4_)2S2O3 + ABTS→ ABTS^•+^ + ArOH→ ABTS + ArO^•^	- permit detection of a wide range of antioxidant compounds, - reacts quickly with synthetic and natural antioxidant agents	- lack of biological relevance, because ABTS radical cation is not found naturally	[32,33,37],
FRAP assay	[Fe^3+^-(TPTZ)_2_]^3+^ + ArOH→[Fe^3+^-(TPTZ)_2_]^2+^ + ArO^•^ + H^+^	- great sensitivity and precision allow it to distinguish between samples, - can test a variety of biological samples such as plasma, blood, serum, saliva, tears and urine	- the tendency of the Prussian blue complex to precipitate and to stain the measuring vessel	[29,33,37]
ORAC assay	R-N=N=R + O_2_→N_2_ + 2ROO^•^ + Fluorescein → Non-fluorescent products	- can be modified for the detection of lipophilic antioxidants	- non-specificity of the fluorescence compounds that can react with other samples, thus losing fluorescence	[29,32]
CUPRAC assay	Cu(Nc_2_)^2+^ + ArOH→Cu(Nc_2_)^+^ + ArO^•^+H^+^	- cheap reagents, more stable and accessible than DPPH or ABTS reagents	- antioxidant enzymes cannot be measurable	[33,37]
HPS assay	C_11_H_13_N_3_O + 2H_2_O_2_ + ArOH→C_11_H_11_N_3_OArO^•^ + 4H_2_O_2_	- is fast and simple	- secondary metabolites found in plants that absorb UV light may cause interference	[36]

DPPH: Diphenyl-1-Picrylhydrazyl; ABTS: 2,2′-Azinobis-(3-Ethylbenozothiazoline-6-Sulfonate; FRAP: Ferric Reducing Antioxidant Power; ORAC: Oxygen Radical Absorbance Capacity; CUPRAC: Cupric Ion Reducing Antioxidant Capacity; HPS: Hydrogen Peroxide.

## Abbreviations

4CL	4-coumaroyl CoA ligase
AAPH	2,2′-azobis(2-amidinopropane) dihydrochloride
ABAP	2,2-azobis(2-amidinopropane) chlorhydrate
ABTS^•+^	2,2-Azinobis 3-ethylbenzthiazoline-6-sulfonic acid radical scavenging
AH^+^	Hydrogen donor
AIBN	α, α, -azobisizobutyronytril
AMVN	2,2′-azobis(2,4-dimethylvaleronytril)
ANCs	Anthocyanins
ArOH	Antioxidant agent
C4H	Cinnamate 4-hydroxylase
CUPRAC	Cupric ions reducing antioxidant capacity
DPPH^•^	2,2-Diphenyl-1-picrylhydrazyl radical scavenging
FRAC	Ferric ion reducing antioxidant power
H_2_O_2_	Hydrogen peroxide
HAT	Hydrogen atom transfer
HRP	Horseradish peroxidase
O/W	Oil in water emulsion
O/W/O	Oil in water in oil double emulsion
ORAC	Oxygen radical absorbance capacity
RNS	Reactive nitrogen species
ROS	Reactive oxygen species
SET	Single electron transfer
TPTZ	Tripyridyltriazine
W/O	Water in oil emulsion
W/O/W	Water in oil in water double emulsion
